# “Updates on diagnostic and prognostic molecular biomarkers of CNS tumors”

**DOI:** 10.1016/j.neurot.2026.e01066

**Published:** 2026-09-17

**Authors:** Shane Eaton, Ahmad Almotairi, Adrian Levine, Cynthia Hawkins

**Affiliations:** a Department of Laboratory Medicine and Pathobiology, University of Toronto, Toronto, ON, Canada; b Division of Pathology, Department of Paediatric Laboratory Medicine, The Hospital for Sick Children, Toronto, ON, Canada; c Laboratory Medicine Program, Department of Pathology, University Health Network, Toronto, ON, Canada; d Department of Pathology, College of Medicine, King Saud University, Riyadh, Kingdom of Saudi Arabia; e The Arthur and Sonia Labatt Brain Tumour Research Centre, The Hospital for Sick Children, Toronto, ON, Canada

**Keywords:** Glioma, Ependymoma, Meningioma, Embryonal, Pineal, MMRD

## Abstract

The diagnosis and classification of central nervous system (CNS) tumors has undergone a paradigm shift over the past decade, evolving from a purely histology-based approach to an integrated framework that incorporates molecular and epigenetic features. This review summarizes recent updates in key genomic and epigenomic biomarkers across major CNS tumor categories, with a focus on their diagnostic, prognostic, and therapeutic implications. DNA methylation profiling has emerged as a valuable tool for tumor classification, subgrouping, and grading, complementing traditional histopathologic assessment. Across diffuse gliomas, newly characterized molecular alterations have refined grading criteria and clarified the boundaries between tumor types, including important caveats about the use of individual molecular features as sole diagnostic criteria. In ependymomas, medulloblastomas, atypical teratoid/rhabdoid tumors, meningiomas, pineal tumors, and embryonal tumors, methylation profiling now defines biologically and clinically meaningful subgroups that inform risk stratification and treatment selection. The emerging recognition of mismatch repair-deficient gliomas and fusion-driven tumor entities further underscores the expanding complexity of CNS tumor taxonomy. As molecular technologies continue to advance, the integration of genomic, epigenomic, histopathologic, and clinical data will be essential to improving diagnostic precision, guiding therapy, and ultimately enhancing patient outcomes.

## Introduction

The diagnosis of central nervous system (CNS) tumors has undergone a major shift in the past decade, from being based entirely on histology, to an integrated approach that includes pathology and molecular features. Advances in molecular testing have demonstrated that tumors with similar histological appearances can have distinct molecular features, which affect prognosis and therapy. Thus, molecular tests play a critical role in tumor classification, prognosis, and management plans. The two recent World Health Organization (WHO) updates, 2016 [1] and 2021 [2], included molecular biomarkers in routine diagnostic criteria, such as *IDH1/IDH2* mutations, 1p/19q codeletion, *CDKN2A/B* deletion, *BRAF* alteration, *EGFR* amplification, *TERT* promoter mutations, and histone H3 alteration. In addition to that, genome-wide DNA methylation has emerged as a useful tool for tumor classification. Due to the rapid advances in this field, the recent WHO updates and Consortium to Inform Molecular and Practical Approaches to CNS Tumor Taxonomy (cIMPACT-NOW), have provided CNS tumor classification and grading updates. The recent updates have revised diagnostic criteria for some entities, updated molecular-based grading features, and clarified the role of methylation for tumor classification. This review summarizes recent updates in key biomarkers (summarized in Table 1), focusing on genomic and epigenetic alterations and their implications on diagnosis and prognosis [[2], [3], [4], [5], [6], [7], [8], [9]].

**Table 1:** Summary of recent updates to molecular biomarkers in CNS tumors.

Tumor type	Genetic alteration	Methylation class	Clinical significance	References
Astrocytoma, IDH-mutant	*PDGFRA* amplification		Emerging molecular marker most consistent with CNS WHO grade 4 behavior	[8,10]
	*PIK3CA/PIK3R1* mutations, *MYCN* amplification, *EGFR* amplification, *CDK4/CDK6* amplification, *CCND2* amplification, *RB1* alteration, *CDKN2A* mutation		Emerging molecular markers that may support CNS WHO grade 3 classification	[8,10]
		Astrocytoma, IDH mutant; high grade/G-cimp-low	Methylation-based high-risk signature	[8]
IDH-wildtype low-grade morphology glioma	Isolated *TERT* promoter mutation		Should not be considered independently diagnostic of glioblastoma CNS WHO grade 4 tumor.	[7,11,12]
Diffuse high-grade glioma/pediatric-type glioma differential	*EGFR* amplification and +7/−10		May occur in other glioma types, so clinical, morphology, location, and methylation should be considered	[7]
ZFTA fusion ependymoma	*CDKN2A/B* loss		Associated with poorer prognosis	[2,7,13]
Posterior fossa ependymoma, PFA	Loss of H3 K27me3		Surrogate marker for PFA ependymoma	[2,14]
	*EZHIP* expression		Aids diagnosis, especially when H3 K27me3 staining is equivocal	[7,15]
	6q loss		Worse prognosis	[16]
	1q gain		Worse prognosis	[13,16]
Posterior fossa ependymal tumor/PFSE methylation class	*TERT* promoter mutation and/or chromosome 6 loss		Supports descriptive NEC designation in certain PFSE-classified tumors	[7,17]
Spinal ependymoma	*MYCN* amplification		Diagnostic criterion for *spinal ependymoma, MYCN-amplified*	[18]
	*NF2* bi-allelic inactivation (subtype A)		Associated with higher risk of recurrence or progression	[19]
Myxopapillary ependymoma		Myxopapillary ependymoma	Aids diagnosis for unresolved lesions	[20]
Subependymoma	*TERT* promoter mutation and/or chromosome 6 loss		May evolve to a higher risk tumor with ependymoma-like histology	[17]
Meningioma	1p loss with 22q loss or *NF2* alteration		Supports upgrading to CNS WHO grade 2	[6]
	≥2 or more whole arm losses on chromosomes 6, 10, 14, or 18		Molecular high risk. Aids in resolution of equivocal histologic grade.	[6]
	*SMARCE1*		Associated with specific histologic subtypes, clear cell meningioma	[6]
	*BAP1* alterations		Associated with specific histologic subtypes, rhabdoid meningioma	[6]
	*TERT* promoter mutation		Molecular criteria for CNS WHO grade 3	[2,6]
	*CDKN2A/B* homozygous deletion		Molecular criteria for CNS WHO grade 3	[2,6]
		Meningioma, subtype benign/intermediate/malignant	Risk stratification	[6]
Medulloblastoma	*MYC* or *MYCN* amplifications		Associated with poorer prognosis in group 3/4	[21]
		Subclass II/III/V	Associated with aggressive behavior and poor survival	[[22], [23], [24]]
		Subclass VIII	Associated with recurrence	[[22], [23], [24]]
Atypical teratoid/rhabdoid tumor		ATRT-TYR	Associated with reduced overall survival compared to non-TYR methylation classes	[25]
Cribriform neuroepithelial tumor		ATRT-TYR	Provisional CNS WHO diagnosis that frequently matches with ATRT-TYR methylation class. Cribriform histology distinguishes CRINET from AT/RT. Prognosis is more favorable than AT/RT.	[26]
CNS neuroblastoma, FOXR2-activated		CNS neuroblastoma, FORXR2-activated	Aids diagnosis for unresolved lesions	[27]
CNS tumor with BCOR internal tandem duplication	*BCOR* internal tandem duplication		Diagnostic criterion	[28]
	*EP300*::*BCOR* fusion or other *BCOR*/*BCORL1* fusions		Emerging as distinct diagnoses within the family of BCOR-altered CNS tumors	[28]
Embryonal tumor with multilayered rosettes	*C1*9MC alteration		Diagnostic criterion	[29]
	*DICER1* alteration		Diagnostic criterion. Suggests overlap with tumor predisposition syndromes	[29]
Pineal tumors	KBTBD4 alteration		Frequent driver mutation in PPTID	[30]
	DICER1 alteration		Predisposition to developing pineoblastoma and, to a lesser extent, PPTID	[30]
		PB-miRNA1, PB-miRNA2	Greater survival compared to non-miRNA subgroups of pineoblastoma (PB-RB1, PB-MYC/FOXR2)	[31]
		PPTID-A, PPTID-B	Subgroups of PPTID with uncertain prognostic significance	[30,31]

### DNA methylation profiling

**Fig. 1:**
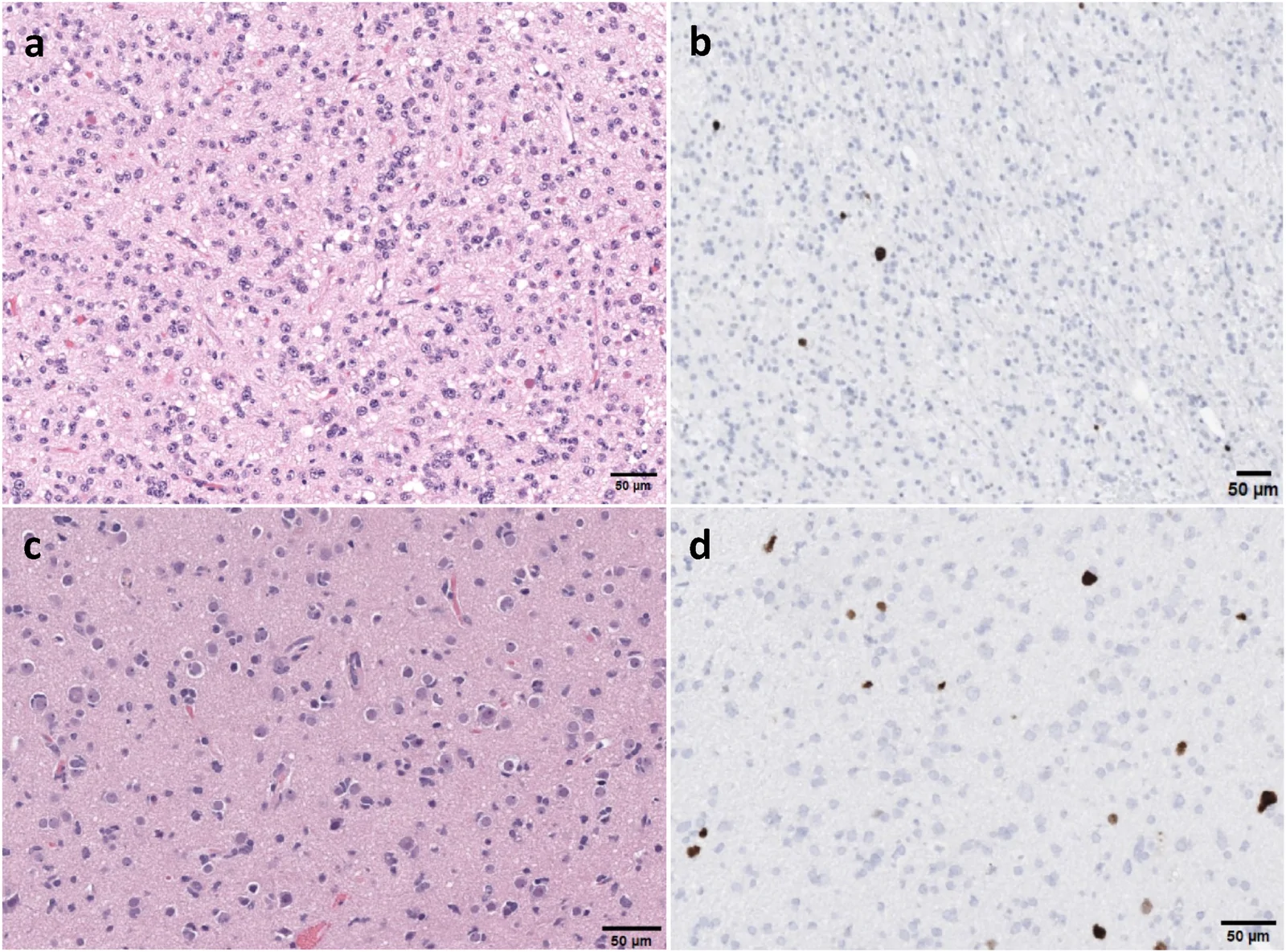
Two illustrative cases demonstrating how molecular biomarkers can help distinguish histologically similar tumors with major clinical differences. (a) Temporal lobe mass in a 21-year-old female demonstrating no high-grade histologic features (hematoxylin and eosin stain). (b) Low Ki-67 proliferative index; DNA methylation profiling classified the tumor within the low-grade ganglioglial/neuroepithelial tumor family (DKFZ v12.8 calibrated score 0.85); no chromosomal alterations were found. (c) Frontal lobe mass in a 34-year-old patient showing a diffuse glioma with no high-grade histologic features (d) Low Ki-67 proliferative index. DNA methylation profiling classified the tumor as adult-type diffuse high-grade glioma, subtype B (DKFZ v12.8 calibrated score 0.97); multiple chromosomal alterations were also detected.

DNA methylation profiling has become important in the diagnosis of several entities. In some cases, it is considered an essential diagnostic criterion, such as for *diffuse glioneuronal tumor with oligodendroglioma-like features and nuclear clusters*, and for many other entities it can resolve diagnostic uncertainty (Fig. 1). In medulloblastoma and posterior fossa ependymoma, it can define subclasses with major clinical implications. Although many cases of methylation class match with WHO tumor classifications, some do not, such as: “adult-type diffuse high-grade glioma, *IDH*-wildtype, subtype F″, and “glioneuronal tumor with *ATRX* alteration, kinase fusion, and anaplastic features”. The use of methylation for grading/prognostication is generally less straightforward than for diagnosis, although it may be relevant in some tumors like meningioma. Despite these advantages, DNA methylation has some limitations, including poor classification accuracy for certain types of entities and unclear clinical relevance for some entities/subtypes. Accordingly, as with all clinical and diagnostic tests, DNA methylation must be interpreted in the overall context of clinical, pathologic, and genetic findings [[3], [4], [5], [6],^9^,[32], [33], [34], [35], [36]].

### Astrocytoma, IDH-mutant

The grading of *astrocytoma, IDH-mutant* has become increasingly important with the recent success of *IDH* inhibitors in clinical trials [37] and use of grade to determine candidates for this treatment. Per WHO CNS5, *astrocytoma IDH-mutant* is graded from 2 to 4, with grade 3 based on histologic criteria (“considerable mitotic activity and histologic anaplasia”), and grade 4 either by histologic features (microvascular proliferation and/or necrosis) or *CDKN2A/B* homozygous deletion [2]. Recent updates, summarized in cIMPACT-NOW update 12, have suggested that a number of additional molecular alterations are associated with worse outcomes and warrant increased grade.

- *PDGFRA* amplification is associated with worse outcomes and is consistent with CNS WHO grade 4.
- *PIK3CA/PIK3R1* mutations, *MYCN* amplification, *EGFR* amplification, *CCND2* amplification, *CDK4/CDK6* amplification, *RB1* alteration, and *CDKN2A* mutation, are indicative of intermediate risk and may support CNS WHO grade 3 classification.
- Methylation class “G-CIMP-low” and “Astrocytoma, IDH mutant; high grade (A_IDH_HG)” are not in keeping with CNS WHO grade 2 and are more aligned with CNS WHO grade 3 or grade 4 based on histology or other molecular features.

Further, this update has provided quantitative histologic criteria for grading, suggesting the use of ≥3 mitoses per 2.4 mm^2^ as a cutoff to differentiate between grade 2 and grade 3 *IDH*-mutant astrocytoma, which helps resolve a major point of uncertainty [8,10].

### Diffuse high-grade gliomas

Glioblastoma (GBM) has traditionally been defined as a high-grade astrocytic neoplasm with microvascular proliferation and necrosis. Recognizing that the histologic appearance can be variable, and some samples (particularly small biopsies) may not show these classic histologic features, in WHO CNS5 the definition of *glioblastoma, IDH-wildtype* was expanded to also include IDH and H3-wildtype diffuse astrocytic gliomas with lower grade histology that have *TERT* promoter mutation, concurrent chromosome 7 gain and chromosome 10 loss (+7/-10), and/or *EGFR* amplification [2]. However, there is increasing recognition that these molecular features are not entirely specific, and that one needs to be cautious about their use to support a diagnosis of *Glioblastoma, IDH-wildtype*, particularly in younger patients (age <40)—recommendations that are summarized in cIMPACT-NOW update 11 [7].

Gliomas with low-grade morphology and isolated *TERT* promoter mutation but without *EGFR* amplification or +7/-10 alteration shows better prognosis than typical *glioblastoma, IDH-wildtype* [7,11,12]. Some of these tumors, particularly those with a gliomatosis cerebri-like growth pattern belong to a distinct methylation class termed “adult-type diffuse high-grade glioma, *IDH*-wildtype, subtype F” [38]. *TERT* promoter mutations have further been reported in a number of other entities such as *pleomorphic xanthoastrocytoma, subependymoma, diffuse pediatric-type high-grade glioma,* and *diffuse gliomas with FGFR3::TACC3 fusion* [[39], [40], [41]]. Therefore it is recommended not to use TERT promoter mutation as the solitary defining feature for GBM in histologically low-grade tumors [7].

Similarly, chromosome +7/-10 has been reported in *pleomorphic xanthoastrocytomas* and *diffuse hemispheric gliomas, H3 G34-mutant*, and EGFR amplification can occur in *diffuse pediatric-type high-grade glioma, H3-wildtype and IDH-wildtype* and *diffuse hemispheric glioma, H3 G34-mutant*. Therefore it is recommended that either EGFR amplification or chromosome +7/-10 should not be used as solitary defining features for GBM in patients under 40 years of age. In uncertain cases further workup with methylation profiling and NGS to rule out BRAF and other RAS-MAPK pathway alterations can be helpful to confirm the diagnosis [7].

It has also been increasingly recognized that IDH and H3-wildtype diffuse high-grade gliomas are biologically different in children and adults, leading to the new entity of *diffuse pediatric-type high-grade glioma, H3-wildtype and IDH-wildtype* (DPHGG) [2]. However it is also apparent that the age ranges of pediatric and adult-type tumors have significant overlap and that pediatric-type tumors can occur in adults up to age 70 [42]. Currently DNA methylation profiling is the most robust way of making a diagnosis of *diffuse pediatric-type high-grade glioma, H3-wildtype and IDH-wildtype*, although the presence of key molecular features such as *PDGFRA* alteration, *EGFR* alteration, or *MYCN* amplification can be used to support the diagnosis in patients under 25 years [7].

### Mismatch-repair deficient (MMRD) gliomas

While not a formal WHO diagnostic entity yet, there is increasing recognition that a subset of diffuse gliomas are driven by primary mismatch repair deficiency, particularly in children, adolescents, and young adults [[43], [44], [45]]. These tumors usually present with high-grade histologic features and loss of one or more components of the mismatch-repair (MMR) complex (*MLH1*, *MSH2*, *MSH6*, *PMS2*) on immunohistochemistry, either in tumor cells alone (Lynch syndrome) or in all tumor and normal cells (constitutional mismatch repair deficiency; CMMRD) [44,45].

Importantly a small proportion will retain expression of the dysfunctional MMR protein, and in these cases additional sequencing can be useful to identify (1) a pathogenic mutation in an MMR gene, (2) elevated tumor mutation burden, and/or (3) mutational signatures of underlying MMRD and microsatellite instability [[44], [45], [46]]. They also usually cluster with the methylation group “diffuse pediatric-type HGG, subtype RTK1A”.

Genetically, MMRD gliomas are virtually always wildtype for *BRAF* and *histone H3*, however a subset do have IDH1 R132H, often with accompanying *TP53* and *ATRX* mutations (and lacking 1p/19q codeletion). Primary mismatch repair–deficient IDH-mutant astrocytoma behaves aggressively, and is associated with poor clinical outcomes, relative to both conventional *astrocytoma, IDH-mutant*, and to IDH-wildtype MMRD HGG [43]. Importantly, tumors with primary MMRD are distinct from those in which it arises secondary to temozolomide treatment [47].

The resulting hypermutated phenotype has an important role in tumor development and progression and also shapes the tumor immune microenvironment, with critical clinical implications [44,46]. MMRD gliomas often have increased immunogenicity and may have dramatic responses to immune checkpoint inhibitors, but generally respond poorly to conventional alkylating chemotherapy [[44], [45], [46]]. Accordingly, it is crucial to identify patients with MMRD HGG, both for the immediate clinical treatment, as well as for implications regarding germline testing and genetic counseling for family members. It is encouraged to test for MMR loss by immunohistochemistry (IHC) in high-grade gliomas in younger patients (age <40) that are wildtype for H3, BRAF, and IDH, as well as in IDH-mutant astrocytomas presenting as high-grade in children, adolescents, and young adult patients (cIMPACT-NOW Update 11 recommends age <18 years [7], although data [45] suggest <25 years is reasonable). In cases where there is high suspicion for MMR loss, but it is not detected by IHC, additional molecular testing by other modalities is recommended, such as next generation sequencing (NGS) for MMRD-associated mutational signatures [48] and/or pathogenic mutations in one of the MMR genes.

### Ependymomas

Ependymal tumors are classified based on morphology (ependymoma, subependymoma, and myxopapillary ependymoma), anatomic location (supratentorial, posterior fossa, and spinal), and molecular features [2,13].

Supratentorial ependymomas have two molecularly defined types in WHO CNS5: *ZFTA* fusion-positive and *YAP1* fusion-positive. *Supratentorial ependymoma, ZFTA fusion-positive* (EPN-ZFTA) is the more common subtype and is typically characterized by *ZFTA::RELA* fusion, although other fusion partners may occur [2,36]. Notably, combined *CDKN2A/B* loss is associated with worse outcome in supratentorial EPN-ZFTA [13]. Rare posterior fossa ependymomas with *ZFTA* fusion have also been reported; these tumors show a similar methylation class and copy-number profile to supratentorial EPN-ZFTA. The recommendation is to report these cases as *posterior fossa ependymoma, ZFTA* fusion-positive, not elsewhere classified (NEC) [7,13]. *Supratentorial ependymoma, YAP1 fusion-positive* is uncommon, occurs predominantly in children, and most frequently harbors a *YAP1::MAMLD1* fusion [2,36].

Posterior fossa (PF) ependymomas are subtyped as group A and B, with DNA methylation profiling as the primary modality. Loss of nuclear H3 K27me3 expression by immunohistochemistry is a surrogate marker for PF group A [2,14], with EZHIP overexpression as another useful marker, which is recommended in ambiguous cases [7,15]. Prognostically, gain of chromosome 1q and loss of chromosome 6q are both associated with worse outcomes, and can be present both at diagnosis or arise in relapsed cases [13,16]. Definitive diagnosis of *posterior fossa group B (PFB) ependymoma* requires DNA methylation profiling. The immunohistochemical profile usually shows retained nuclear H3 K27me3 expression and lack of EZHIP overexpression. However, this immunophenotype is not specific for PFB, because other posterior fossa ependymal tumors, including tumors with a “posterior fossa subependymoma” (PFSE) methylation class, may show the same findings. When methylation profiling is unavailable, a posterior fossa ependymoma with retained H3 K27me3 expression and absent EZHIP overexpression should be diagnosed as *posterior fossa ependymoma, not otherwise specified (NOS)* [2,7,15,36]. Tumors with pure ependymoma morphology or mixed ependymoma-subependymoma morphology that are classified as subependymoma by methylation profiling remain unclear in the CNS5 classification. Many of these tumors harbor *TERT* promoter mutations and chromosome 6 loss. The descriptive label “posterior fossa ependymal tumor with *TERT* promoter mutation and/or chromosome 6 loss, with PFSE methylation class assignment, NEC” has been recommended [7,17].

For spinal ependymomas, epigenetic profiling with transcriptomic analysis has identified two distinct subtypes within the spinal ependymoma methylation class: subtype A and B. Subtype A is characterized by bi-allelic *NF2* inactivation, typically occurring via concurrent chromosome 22q loss and a somatic or germline *NF2* mutation. This subtype shows reduced *NF2* expression, increased nuclear pleomorphism, and a more aggressive clinical phenotype with a higher risk of recurrence or progression. In contrast, Subtype B tumors typically demonstrate monoallelic *NF2* loss with retained *NF2* expression and more extensive global copy-number alterations [19,49,50].

WHO CNS5 recognizes *spinal ependymoma, MYCN-amplified* as a distinct, highly aggressive molecular entity. The pathognomonic biomarker is high-level amplification of the *MYCN* proto-oncogene (chromosome 2p24.3), which remains stable at relapse. These tumors frequently harbor additional chromosomal copy-number alterations, most commonly loss of chromosome 10q and focal losses on 11q. Clinically, *MYCN*-amplified spinal ependymomas are characterized by extramedullary growth, multi-segmental spinal involvement, early leptomeningeal dissemination, and resistance to conventional therapies. Potential therapeutic strategies targeting the *MYCN* driver are under investigation, including inhibitors of *HDAC*, *PARP*, Aurora A-kinase, and BET-bromodomain proteins [[49], [50], [51]].

*Myxopapillary ependymomas* (MPE) are classified as WHO grade 2 in CNS5 due to rates of recurrence and thecal sac seeding. They have a distinct methylation class, at least partially driven by differential CpG site hypomethylation at the *HOXB13* locus. HOXB13 IHC is emerging as an effective nuclear marker to distinguish MPE from classic spinal ependymoma and other histological mimics [52]. Genetically, MPE frequently exhibits gains of chromosome 16 and losses of chromosome 10. These tumors can also exhibit a metabolic shift toward a Warburg phenotype, demonstrated by the upregulation of *HK2*, *PKM2* and *PDK* [53].

Subependymomas are indolent CNS WHO grade 1 tumors that, while morphologically similar across locations, exhibit distinct DNA methylation profiles depending on their anatomical compartment (supratentorial, posterior fossa, or spinal). Copy number alterations seen in subependymomas include chromosome 19 loss and partial chromosome 6 loss. While most subependymomas follow an indolent course, a small subset of posterior fossa subependymomas harboring chromosome 6 loss or *TERT* promoter mutations have been linked to evolution to a higher risk tumor with ependymoma-like histomorphology [17].

### Meningiomas

Recent advances have shifted meningioma classification from a predominantly histologic approach toward an integrated morphologic and molecular framework. The WHO CNS5 incorporated select molecular alterations into grading – specifically, *TERT* promoter mutations and homozygous deletion of *CDKN2A/B* were assigned CNS WHO grade 3, irrespective of histologic features, reflecting their association with aggressive clinical behavior. However, mounting evidence suggests TERT-promoter mutation alone in histologically low grade meningiomas does not equate to high grade behavior and should be interpreted cautiously [54]. (Note that current evidence does not support the use of hemizygous *CDKN2A/B* loss for grading).

Molecular studies have identified biologically distinct subgroups. *NF2*-altered meningiomas are more commonly located in the convexity and are associated with chromosomal instability and a higher risk of recurrence. In contrast, non-*NF2* tumors, characterized by mutations in genes such as *TRAF7*, *AKT1*, *KLF4*, and *SMO*, typically arise in the skull base and are more often associated with lower-grade behavior. Certain mutations correlate with specific histologic variants, including *SMARCE1* in clear cell meningioma and *BAP1* alterations in rhabdoid tumors [6].

cIMPACT-NOW 8 has substantially expanded the recommendations regarding molecular grading criteria [6], which will likely be highly useful in histologically borderline or ambiguous cases between grade 1–2. Brain invasion remains a criterion for CNS WHO grade 2, however, its assessment may be challenging and subject to overinterpretation. In cases where invasion is equivocal, particularly in those that are otherwise histologically grade 1 (termed brain-invasive otherwise benign meningiomas; BIOB), molecular data should be incorporated before final grading. Tumors with borderline mitotic activity, unexpected clinical behavior, or higher-risk histologic subtypes such as chordoid, clear cell, papillary, or rhabdoid should also undergo additional molecular evaluation. When molecular testing is not available, designation as NOS may be appropriate.

Recurrent chromosomal copy number alterations provide additional prognostic information. Loss of chromosome 22q, involving the *NF2* locus, is the most frequent alteration and represents an early event in tumorigenesis. Deletion of chromosome 1p is associated with increased risk of recurrence and progression. The combination of 1p loss with 22q loss or an *NF2* alteration supports upgrading an otherwise low-grade tumor to CNS WHO grade 2, as does the presence of two or more whole arm losses on chromosomes 6, 10, 14, and 18. Additional alterations, including losses of 14q and 9p, the latter involving *CDKN2A/B*, are associated with higher-grade disease and increased genomic instability [6].

While meningiomas are common among adults, they are notably less frequent in the pediatric (age 0–14) and adolescent/young adult (age 15–39) age groups. Due to the limited data on meningiomas in younger populations, molecular prognostication has not been as robust as in adult meningiomas and should be interpreted with caution [55,56].

DNA methylation profiles can also provide prognostic subgrouping that can suggest higher-grade behavior, although it can be challenging to interpret these when different classifier versions have discordant results with each other or with CNV-based grading. Ultimately, integrated models that combine histologic features, copy number alterations, and methylation class can likely provide improved prognostic accuracy and identify tumors at higher risk of aggressive behavior. Importantly, these new recommendations should only be applied to sporadic adult meningiomas, as pediatric, *NF2*-associated, and radiation-induced meningiomas are not well represented in current studies and require further investigation.

### Medulloblastomas

The contemporary management of medulloblastoma has transitioned into a highly stratified paradigm in which multiomic profiling has defined distinct biological entities with vastly different clinical trajectories. The *WNT*-activated subgroup is primarily defined by *CTNNB1* mutations and monosomy 6, exhibiting a remarkably favorable prognosis with nearly 100% five-year survival, which has made these patients primary candidates for treatment de-escalation research [57]. In contrast, the *SHH*-activated group is divided into *TP53-*mutant and *TP53-*wildtype. Those with *TP53* mutations show therapeutic resistance and significantly lower survival rates compared to wildtype. Group 3 and Group 4 tumors are further refined through DNA methylation into eight distinct subclasses (I-VIII) that provide granular prognostication [21,22].

Subclasses II, III, and V comprise a high-risk group that demonstrate aggressive behavior, metastatic dissemination, and poor overall survival. Subclass II is characterized by *MYC* amplifications and a high prevalence of large-cell/anaplastic histology, often accompanied by cytogenetic gains of chromosomes 1q, 8, and 13q. Subclass III shows focal *MYC* or *MYCN* alterations alongside chromosomal deletions on 8p and 10q. Subclass V is frequently metastatic and shows concurrent *MYC* and *MYCN* amplifications, accompanied by isochromosome 17q and deletions on 16q.

Subclasses I, IV, VI, and VII comprise a more favorable risk group. Subclass I exhibits a largely balanced genome with localized *OTX2* amplifications. Subclass IV is predominantly diagnosed in infants and displays frequent losses of chromosomes 8, 10, 11, and 13. Subclasses VI and VII frequently align with the whole-chromosomal aberration favorable-risk phenotype (gains of chromosome 7 and losses of 8 and 11); Subclass VI typically features *PRDM6* enhancer hijacking, whereas Subclass VII is defined by somatic *KBTBD4* mutations.

Subclass VIII constitutes a unique late-relapse risk group. Predominantly presenting in older children, these tumors exhibit a quiet genome where isochromosome 17q is often the sole cytogenetic aberration, alongside mutations in chromatin remodeling genes like *KDM6A*. Although patients with Subclass VIII tumors initially demonstrate favorable survival comparable to standard-risk cohorts, they remain prone to disease recurrence five or more years post-diagnosis [[22], [23], [24]].

### Other CNS embryonal tumors

*Atypical teratoid/rhabdoid tumors (ATRT)* are typically defined by the biallelic inactivation of the *SMARCB1* gene or less commonly the *SMARCA4* gene. They have been subdivided into 3 molecular subgroups by methylation profiling: ATRT-TYR, ATRT-SHH, and ATRT-MYC [58]. The ATRT-TYR subgroup has the most favorable overall survival among the three subgroups, with a reported 5-year overall survival rate of approximately 48% compared to 24% in non-TYR subgroups [25]. A non-TYR profile thus serves as a negative prognostic indicator.

The ATRT-TYR subgroup is characterized by the upregulation of melanosomal genes, most notably tyrosinase (*TYR*), *MITF*, and *DCT*. Additional biomarkers include the developmental transcription factor *OTX2* and components of the BMP signaling pathway such as *BMP4*. The loss of *SMARCB1* in this subgroup is typically via the whole or partial loss of one copy of chromosome 22 accompanied by an inactivating mutation on the remaining allele. Epigenetically, ATRT-TYR exhibits global DNA hypermethylation [59,60]. The median age at diagnosis of ATRT-TYR is approximately 12 months.

The ATRT-SHH subgroup is characterized by the activation of Sonic Hedgehog (*SHH*) signaling (e.g., *GLI2*, *BOC*, *PTCHD2*, *MYCN*) and Notch signaling pathways (e.g., *ASCL1*, *HES1*, *DLL1/3*). The loss of *SMARCB1* in this subgroup is typically compound heterozygous point mutations rather than broad chromosomal deletions. Similar to ATRT-TYR, this subgroup exhibits a globally hypermethylated epigenetic profile [24,58]. The median age of diagnosis of ATRT/SHH is approximately 20 months and are found at roughly equal frequencies in both supratentorial and infratentorial locations.

The ATRT-MYC subgroup is characterized by high expression of *MYC*, *HOXC* cluster genes, and the long non-coding RNA *HOTAIR*. Unlike the other subgroups, ATRT-MYC tumors are hypomethylated. Loss of *SMARCB1* in this subgroup is typically marked by focal homozygous deletions of *SMARCB1* [59,60]. This subgroup has a median age of diagnosis of 27 months. While they are primarily supratentorial, the ATRT-MYC subgroup notably accounted for all spinal tumors observed in a consensus cohort [58]. Interestingly, ATRT-MYC tumors exhibit the highest levels of cytotoxic T-cell infiltration and immune checkpoint expression (PD-L1/PD-1), suggesting they may be the most suitable candidates for immunotherapy approaches [59,61].

*Cribriform neuroepithelial tumor (CRINET)* is a provisional CNS WHO entity. It is a *SMARCB1*-altered, INI1-deficient tumor that shares molecular similarities with ATRT-TYR but lacks overt rhabdoid morphology and shows characteristic cribriform neuroepithelial architecture. Compared with classic ATRT, CRINET appears to be associated with significantly better long-term survival, making its distinction from ATRT clinically meaningful [26].

*CNS neuroblastoma, FOXR2-activated*, is defined by activation of the *FOXR2* transcription factor, most often through structural rearrangements that may include insertions, inversions, duplications, deletions, or more complex rearrangements. These tumors frequently show recurrent copy-number alterations, including chromosome 1q gain, and may also demonstrate 16q loss, 11q loss, chromosome 8 gain, 17q gain, and X chromosome loss in females [27]. This diagnosis is suggested histologically by a tumor with embryonal morphology (sheets of poorly differentiated cells), sometimes with neurocytic or ganglion cell morphology, and that is positive for OLIG2 by IHC, which distinguishes it from most other embryonal tumors.

*CNS tumor with BCOR internal tandem duplication* is an aggressive neuroepithelial tumor defined by an in-frame internal tandem duplication in exon 15 of *BCOR*. These tumors often occur in children and may show high-grade histologic features, including necrosis, microvascular proliferation, and primitive or ependymoma-like morphology. Precise molecular identification is important because they can resemble anaplastic ependymoma, high-grade glioma, or embryonal tumor NOS. While the morphology can be variable, a clue to this diagnosis is the lack of IHC positivity to OLIG2, GFAP, S100, and neuronal markers, which can help exclude much of the differential diagnosis. Positivity for BCOR IHC is a sensitive marker but is not specific and can occur in other CNS tumors, such as solitary fibrous tumor. More recent work has also expanded the broader family of *BCOR*-altered CNS tumors, including tumors with *EP300::BCOR* and other *BCOR/BCORL1* fusions, which appear related but should not be conflated with the WHO-defined *BCOR-ITD* entity [28].

*Embryonal tumor with multilayered rosettes* (ETMR) has also undergone further molecular refinement. Most classic ETMRs are characterized by C19MC amplification or structural activation, often associated with LIN28A expression, but C19MC-negative ETMR-like tumors are increasingly recognized. Some of these harbor alterations in *DICER1* or other microRNA-processing pathway genes, highlighting overlap with broader embryonal tumor predisposition syndromes and reinforcing the need for methylation profiling and molecular confirmation in morphologically suggestive cases [29].

### Pineal tumors

Developments in tumors of the pineal region mirror this broader trend toward molecular refinement. *Pineal parenchymal tumors of intermediate differentiation* (PPTID) are increasingly recognized as molecularly heterogeneous and can be divided into prognostically relevant methylation subgroups, often referred to as PPTID-A and PPTID-B. *KBTBD4* insertions or mutations have emerged as recurrent alterations in a subset of pineal parenchymal tumors, although their precise diagnostic and prognostic significance continues to be refined [30,62].

Pineoblastoma (PB) has also been molecularly subdivided into biologically distinct groups, including PB-miRNA1, PB-miRNA2, PB-RB1, and PB-MYC/FOXR2, with recurrent alterations involving microRNA-processing genes such as *DICER1*, *DROSHA*, and *DGCR8* in the miRNA-associated subgroups, and *RB1* or *MYC/FOXR2* alterations in other subgroups, which have reduced survival compared to the miRNA-associated subgroups. These molecular findings have important implications for diagnosis, prognosis, and germline predisposition assessment [31].

A critical diagnostic addition in the pineal region is *desmoplastic myxoid tumor of the pineal region, SMARCB1-mutant*. Although this tumor is *SMARCB1*-deficient, it is distinct from *atypical teratoid/rhabdoid tumor* and typically occurs in the pineal region, often in adolescents or adults. Its recognition prevents misclassification as ATRT and supports more anatomically and biologically appropriate risk stratification. In addition, methylation profiling has shown that a subset of *WNT*-activated embryonal tumors arising in the pineal region cluster with *WNT*-activated medulloblastoma, raising the possibility that some represent ectopic medulloblastomas or a distinct *WNT*-activated pineal embryonal tumor subgroup rather than conventional Pineoblastoma [30,62].

### Emerging fusion-driven CNS tumor entities

Several emerging CNS tumor entities are defined by recurrent gene fusions and distinct DNA methylation profiles. *PLAGL1*-fused neuroepithelial tumor is a recently described entity with variable morphology, although most cases show evidence of ependymal differentiation. These tumors commonly harbor *EWSR1::PLAGL1* or *PLAGL1::FOXO1* fusions and should be considered in supratentorial ependymal tumors lacking *ZFTA* or *YAP1* fusions [63,64]. Kinase fusion-driven tumors are also increasingly recognized, including glioneuronal tumor with *ATRX* alteration, kinase fusion, and anaplastic features (GTAKA), which is characterized by *ATRX* alteration and recurrent kinase fusions, most commonly involving *NTRK(1/2/3)* [65]. Other novel fusion-driven entities include *PATZ1*-fused neuroepithelial tumors, which harbor recurrent *MN1::PATZ1* or *EWSR1::PATZ1* fusions and show broad histologic heterogeneity despite forming a distinct methylation class [66]. Additional rare fusion-defined entities include CNS embryonal tumor with *BRD4::LEUTX* fusion and CNS tumor with *EP300::BCOR* fusion [67,68].

## Conclusions

The classification of central nervous system tumors continues to evolve toward an increasingly nuanced, integrated diagnostic paradigm with direct therapeutic relevance. While current molecular testing requires separate methods for NGS and methylation profiling, novel sequencing methods (5-base sequencing, Oxford Nanopore) are able to directly detect 5-methylcytosine (5-mC)—the so-called 5th base, after adenosine (A), guanine (G), thymine (T) and cytosine (C)—without the need for bisulfide conversion. Incorporating these methods in clinical workflows will likely improve turnaround times by allowing detection of methylation profile and genomic alterations in a single test [69,70].

Genomic and DNA methylation profiling have transformed the evaluation of CNS tumors by resolving ambiguous morphologic diagnoses, refining tumor grading, and identifying biologically meaningful subtypes with distinct clinical behavior. While these molecular tools are not available at every institution, when utilized and interpreted alongside histology, immunohistochemistry, imaging, and clinical context, they provide greater diagnostic accuracy for prognostication and treatment selection.

This molecular refinement is also reshaping neuro-oncology by making current multimodal, risk-adapted treatment strategies more precise. Identifying actionable alterations, pathway dependencies, and methylation-defined subgroups allows for better patient stratification, more focused clinical trial design, and the development of targeted, immune-based, and combination therapies matched to tumor biology. New tumor entities and subdivisions continue to be characterized as molecular approaches become more advanced. Future research using multi-omics approaches will further elucidate the biological drivers of CNS tumors, clarify mechanisms of treatment resistance, and identify new therapeutic vulnerabilities. As these discoveries are translated into practice, careful integration of molecular, epigenetic, histopathologic, radiologic, and clinical data will remain essential to ensure that precision neuro-oncology improves diagnostic accuracy, guides appropriate therapy, and ultimately benefits patients.

## Author contributions

Shane Eaton and Ahmad Almotairi contributed equally in writing the manuscript and preparing the tables and figures.

Adrian Levine and Cynthia Hawkins are co-supervising authors who advised and edited the manuscript.

## Declaration of generative AI and AI-assisted technologies in the manuscript preparation process

Statement: During the preparation of this work, the author(s) used Google NotebookLM in order to aid in the drafting of scientific prose from content outlines. After using this tool/service, the author(s) reviewed and edited the content as needed and take(s) full responsibility for the content of the published article.

## Declaration of competing interest

The authors declare that they have no known competing financial interests or personal relationships that could have appeared to influence the work reported in this paper.
